# Is it necessary to cover the macular hole with the inverted internal limiting membrane flap in macular hole surgery? A case report

**DOI:** 10.1186/s12886-015-0104-1

**Published:** 2015-08-26

**Authors:** Chung-yee Chung, David Sai-hung Wong, Kenneth Kai-wang Li

**Affiliations:** Department of Ophthalmology, United Christian Hospital, Room A, 1/F, Block G, 130 Hip Wo Street, Kwun Tong, Kowloon Hong Kong; Department of Ophthalmology, LKS Faculty of Medicine, The University of Hong Kong, Room 301, Level 3, Block B, Cyberport 4, 100 Cyberport Road, Hong Kong, Hong Kong

## Abstract

**Background:**

To report a case of late closure of idiopathic full-thickness macular hole (FTMH) after vitrectomy with the inverted internal limiting membrane (ILM) technique.

**Case presentation:**

A 68-year-old lady with a stage IV FTMH underwent pars plana vitrectomy with 25 gauge plus transconjunctival system, ILM peeling and gas tamponade. The inverted ILM flap technique was adopted, except that no extra surgical manipulation was used to cover the macular hole with the ILM flap. Surgical outcome was monitored with serial optical coherence tomography (OCT).

Complete closure of the FTMH with resolution of intraretinal cystic changes was confirmed on OCT at 16 months postoperatively. Visual acuity improved from a baseline level of 0.1 to 0.4.

**Conclusion:**

Idiopathic macular hole closure could be delayed to beyond 1 year following the inverted ILM flap technique, especially if the macular hole was not covered with the ILM flap. Not all macular holes that fail to close in the early postoperative period need to be re-operated and there may be no risk of further visual deterioration.

## Background

The closure rate of idiopathic macular hole surgery varies from 68 to 98 % and has greatly enhanced since the introduction of internal limiting membrane (ILM) peeling [1–4]. Despite ILM peeling, surgical closure of large idiopathic macular hole remains challenging. Michalewska et al. described their technique of inverted ILM flap in large macular holes with encouraging results [5]. Controversies remain for whether the macular hole should be covered with the ILM flap at the conclusion of vitrectomy. Here we report a case of late closure of idiopathic macular hole after vitrectomy with the inverted ILM flap technique.

## Case presentation

### Patient and operative details

**Fig. 1:**
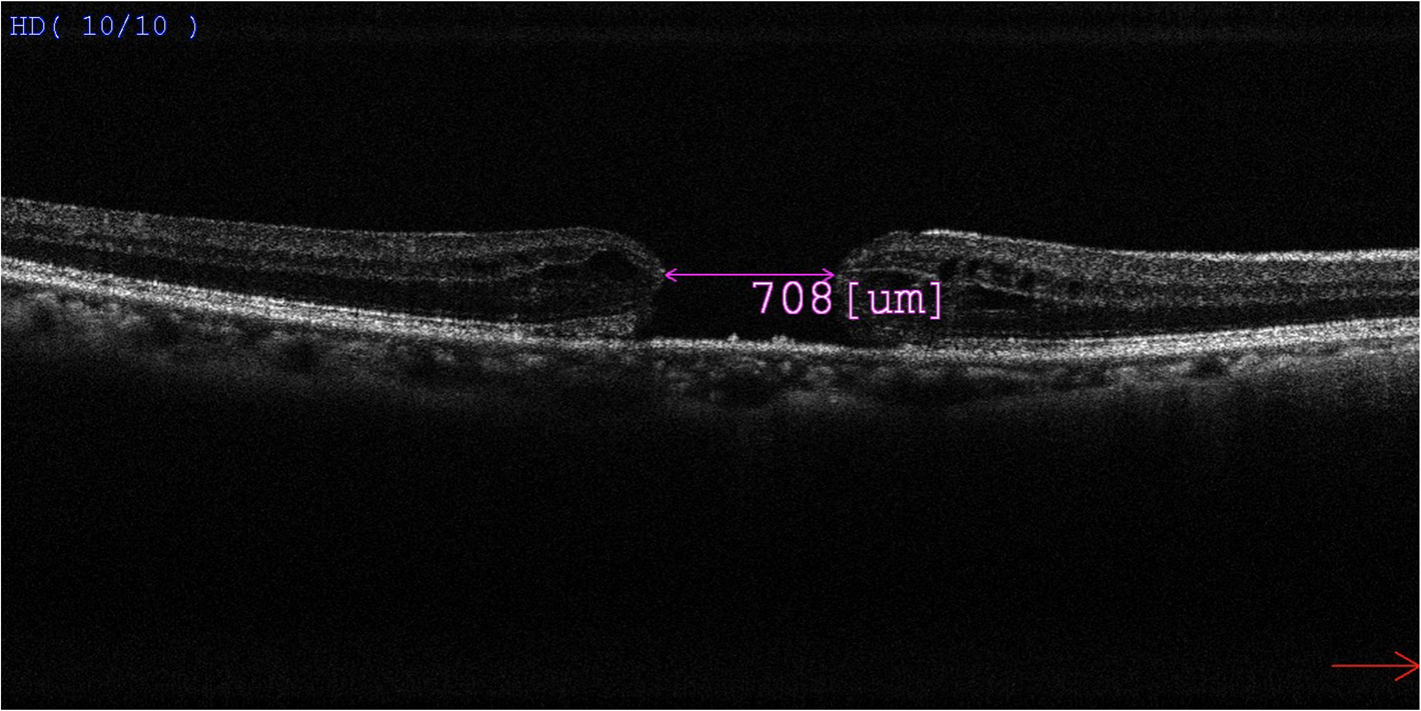
Preoperative Spectral domain-OCT of the full-thickness macular hole, measuring 708 um in basal diameter

A 68-year-old lady with normal tension glaucoma was noticed to have decreased visual acuity of the right eye from 0.5 to 0.1 during routine follow-up. Fundal examination revealed a stage IV full-thickness macular hole in the right eye. Spectral domain optical coherence tomography (OCT RS-3000, Nidek Inc, Gamagori, Aichi, Japan) confirmed the full-thickness nature of the macular hole and the basal diameter of the macular hole was 708 micron (Fig. 1), with the typical cystoid macular oedema over its edges. There was also posterior vitreous detachment.

She was arranged to undergo combined phacoemulsification, intraocular lens implantation, pars plana vitrectomy with 25 gauge plus transconjunctival system (Constellation®, Alcon Inc, Fort Worth, Texas, USA), ILM peeling after staining with MembraneBlue-Dual® (Dutch Ophthalmics Research Centre, Zuidland, The Netherlands) and gas tamponade with 12 % perfluoropropane. The surgical technique was similar to what we described previously [1], except the ILM staining was performed under Balance Salt Solution. In view of the chronicity and large size of the macular hole, the ILM flap technique described by Michalewska et al. was also adopted [5], but no attempt was made to cover the macular hole with the ILM flap. The ILM surgically removed was sent for histological analysis and its presence was confirmed. Postoperatively the patient was asked to adopt a face down posture for 2 weeks. She was prescribed a combination regime of eyedrops of dexamethasone and chloramphenicol (Gutt Nadexin 6 times per day) for 3 weeks. The postoperative progress was monitored with serial visual acuity and spectral domain OCT monitoring.

### Results

**Fig. 2:**
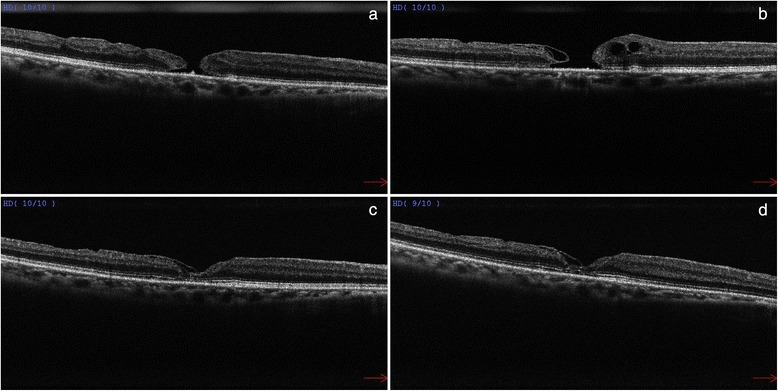
**a** OCT at postoperative 3 months showed a persistent but smaller full-thickness macular hole measuring 200 μm in basal diameter. **b** OCT at postoperative 6 months showed intraretinal cystic changes at the edges of the persistent macular hole. **c** OCT at postoperative 16 months showed complete resolution of cystoid macular oedema with macular hole closure. **d** OCT at postoperative 26 months showed macular hole closure with good delineation of the external limiting membrane and ellipsoid zone

The postoperative period was uneventful and our patient was compliant to posturing. After absorption of the gas bubble, there was a small persistent macular hole measuring 200 μm in diameter on OCT (Fig. 2a). The visual acuity of the right eye improved slightly to 0.15. The option of reoperation with further gas tamponade was discussed but our patient declined any further surgical intervention. The patient continued to be follow-up in our clinic.

During subsequent follow-up OCT was used to monitor her progress. At postoperative 6 months, new intraretinal cystic changes were observed on the edges of the macular hole (Fig. 2b). At postoperative 16 months, there was complete resolution of cystoid macular oedema, and a bridging membrane from the temporal edge leading to closure of the macular hole (Fig. 2c). At postoperative 26 months, her right eye visual acuity was found to improve to 0.4. OCT also showed good delineation of the external limiting membrane and ellipsoid zone, with the bridging membrane from the temporal edge remained in situ (Fig. 2d).

### Discussion

The inverted ILM flap provides both Muller cell fragments to stimulate glial cell proliferation and a basement membrane substrate which acts as a scaffold for tissue proliferation. In the original inverted ILM technique described, the macular hole was covered with the inverted ILM flap [5]. Subsequently, Kuriyama et al. and Shin MK et al. also reported encouraging results in two case series employing a similar technique covering the macular holes with inverted ILM flaps [6, 7].

Whether it is required to cover or tuck the macular hole with the ILM flap remains controversial. According to the OCT images in the original study by Michalewska et al., the ILM flap might have been tucked inside the macular hole but subsequently studies described only covering of the macular hole with the ILM flap was necessary [5–7]. Surgical manipulation to tuck the ILM flap into the hole is not recommended as this can potentially damage the retinal pigmentary epithelium at the base of the macular hole. In the present case, no extra surgical manipulation was used to cover the macular hole with the ILM flap, and it might have led to the initial failure of complete macular hole closure. Although the ILM flap was not detected during the early OCT scans (Fig. 2a, b), the bridging membrane from the temporal edge visualized on later postoperative OCT scans (Fig. 2c, d) might have represented the ILM flap which has turned itself over and covered the hole during the course of observation leading to complete hole closure.

Late closure of idiopathic macular hole closure following surgery is a rare occurrence, although there were isolated reports of late closure of surgically repaired myopic macular holes and traumatic macular holes [8, 9]. Gross et al. reported two cases of reopening and spontaneous closure of previously repaired macular holes with corresponding OCT findings [10]. On serial OCT scans, intraretinal cystic changes on the edges of the macular hole with gradual resolution and spontaneous macular hole closure were observed. Similar observation was also reported by Inoue et al. that included six cases with spontaneous closure of primary macular hole [11]. However for idiopathic macular hole, this is the first reported case of late closure following vitrectomy with the inverted ILM flap technique. We also observed similar morphological changes in the present case over a course of 16 months.

## Conclusion

The present case demonstrated that idiopathic macular hole closure could be delayed to beyond 1 year following the inverted ILM flap technique. In case that no extra surgical manipulation is used to cover the macular hole with the ILM flap during vitrectomy, a longer period of observation might be warranted. Not all macular holes that fail to close in the early postoperative period need to be re-operated and there may be no risk of further visual deterioration.

## Patient consent

Written informed consent was obtained from the patient for publication of this case report and any accompanying images. A copy of the written consent is available for review by the Editor of this journal.

## Availability of supporting data

The serial optical coherence tomography images supporting the results of this article are included within the article and its additional files named Figs. 1 and 2a-d.

## Abbreviations

ILM: Internal limiting membrane
OCT: Optical coherence tomography

## References

[1] Li K, Wong D, Hiscott P (2003). Trypan blue staining of internal limiting membrane and epiretinal membrane during vitrectomy: Visual results and histopathological findings. Br J Ophthalmol.

[2] Kelly NE, Wendel RT (1991). Vitreous surgery for idiopathic macular holes: results of a pilot study. Arch Ophthalmol.

[3] Ando F, Sasano K, Ohba N (2004). Anatomic and visual outcomes after indocyanine green-assisted peeling of the retinal internal limiting membrane in idiopathic macular hole surgery. Am J Ophthalmol.

[4] Beutel J, Dahmen G, Ziegler A (2007). Internal limiting membrane peeling with indocyanine green or trypan blue in macular hole surgery: a randomized trial. Arch Ophthalmol.

[5] MIchalewska Z, Michaelewski J, Adelman RA (2010). Inverted internal limiting membrane flap technique for large macular holes. Ophthalmology.

[6] Kuriyama S, Hayashi H, Jingami Y (2013). Efficacy of inverted ILM flap technique for the treatment of macular hole in high myopia. Am J Ophthalmol.

[7] Shin MK, Park KH, Park SW (2014). Perfluoro-n-octane-assisted single-layered inverted internal limiting membrane flap technique for macular hole surgery. Retina.

[8] Georgalas I, Ezra E (2007). Delayed closure after surgery for a full-thickness macular hole in a highly myopic eye. Can J Ophthalmol.

[9] Rishi P, Reddy S, Rishi E (2012). Delayed, spontaneous conversion of type 2 closure to type 1 closure following surgery for traumatic macular hole associated with submacular hemorrhage. Oman J Ophthalmol.

[10] Gross JG (2005). Late reopening and spontaneous closure of previously repaired macular holes. Am J Ophthalmol.

[11] Inoue M, Arakawa A, Yamane S (2012). Long-term outcome of macular microstructure assessment by optical coherence tomography in eyes with spontaneous resolution of macular hole. Am J Ophthalmol.

